# The reciprocal antagonistic interplay between HIV-1 Tat and SIN3A specifies the epigenetic switch between de-repression versus maintenance of HIV-1 gene silencing

**DOI:** 10.1186/1742-4690-10-S1-O12

**Published:** 2013-09-19

**Authors:** Anurag Kulkarni, Estelle Villemaine, Ann Marie McCartin, Elena Woods, Celine Marban, Virginie Gautier

**Affiliations:** 1 UCD-Centre for Research in Infectious Diseases (CRID), School of Medicine and Medical Science, University College Dublin, Dublin, Ireland; 2 INSERM U1121, Faculté de Chirurgie Dentaire, Université de Strasbourg, Strasbourg, France

## Background

Post-integration latency is a critical barrier to achieving a complete cure for HIV. Epigenetic micro-environment alterations at the integrated HIV-1 LTR, coupled with modulations of the HIV-1 Tat feedback circuit, influence the onset and maintenance of latency. In this context, we have previously identified the epigenetic core pressor SIN3/HDAC complex as part of the *in-vitro* HIV-1 Tat nuclear interactome. Here we delineate the dynamics of their antagonistic functional relationship in the control of HIV-1 latency.

## Methods

Well-studied T-cellular models of HIV-1 latency viz. J-LAT A1 & A72, with latent integrated LTR-Tat-IRES-GFP-LTR and LTR-GFP-LTR cassettes respectively, and ACH-2, with latent full-length integrated HIV-1 provirus in their genome, were employed. We used ChIP-qPCR to study protein occupancy at the HIV-1 LTR; and RT-qPCR and p24 ELISA/GFP FACS to concurrently monitor the reactivation of HIV-1 gene expression. SIN3A knock-down was performed with shRNA targeting sin3a or non-target control using Amaxa nucleofection, and was validated by WB and RT-PCR analysis. Immunofluorescence microscopy was performed in Hela cells co-transfected with Tat and Sin3a.

## Results

The latent HIV-1 LTR displays a constitutive enrichment of the SIN3/HDAC complex.

ChIP-qPCR revealed that the SIN3/HDAC key components SIN3A, SAP18, Sap3O and HDAC-1 were enriched in ACH-2, J-LAT A1 and A72 cells. Importantly, a key event in HIV-1 reactivation from latency is the displacement of the SIN3/HDAC complex from the HIV-1 LTR, as TNFα (NF-KB activator), 5-AZA (DNA methylation inhibitor) or SAHA (HDAC inhibitor) treatments disrupted SIN3/HDAC association with the HIV-1 LTR in latently infected cells.

SIN3A is critical for the maintenance of HIV-1 post-integration latency. SIN3A depletion in J-LAT A1 and A72 resulted in HIV-1 reactivation from latency, which was associated with the depletion of SIN3A and concurrent recruitment of POLII and/or HIV-1 Tat, at the HIV-1 LTR. Next we combined SIN3A knock-down with various reactivation treatments and observed that SIN3A depletion potentiated proviral reactivation by TNFα, PMA/ionomycin (Protein Kinase C activator), 5-AZA and SAHA in J-LAT A1 cells. Thus when associated with the HIV-1 LTR, SIN3A participates in the silencing of the provirus.

